# Examining the Delivery of a Tailored Chinese Mind-Body Exercise to Low-Income Community-Dwelling Older Latino Individuals for Healthy Aging: Feasibility and Acceptability Study

**DOI:** 10.2196/40046

**Published:** 2022-09-13

**Authors:** Yan Du, Neela Patel, Arthur Hernandez, Maria Zamudio-Samano, Shiyu Li, Tianou Zhang, Roman Fernandez, Byeong Yeob Choi, William M Land, Sarah Ullevig, Vanessa Estrada Coats, Jessh Mondesir Mavoungou Moussavou, Deborah Parra-Medina, Zenong Yin

**Affiliations:** 1 Center on Smart and Connected Health Technology School of Nursing University of Texas Health Science Center at San Antonio San Antonio, TX United States; 2 Department of Family and Community Medicine School of Medicine University of Texas Health Science Center at San Antonio San Antonio, TX United States; 3 Dreeben School of Education University of the Incarnate Word San Antonio, TX United States; 4 Alamo Colleges District-North Vista College San Antonio, TX United States; 5 Department of Kinesiology University of Texas at San Antonio San Antonio, TX United States; 6 Department of Population Health Science School of Medicine University of Texas Health Science Center at San Antonio San Antonio, TX United States; 7 College for Health, Community and Policy University of Texas at San Antonio San Antonio, TX United States; 8 Department of Public Health University of Texas at San Antonio San Antonio, TX United States; 9 Latino Research Institute Latino Studies University of Texas at Austin Austin, TX United States

**Keywords:** qigong, mind-body exercise, Five Animal Frolics, health technology, older adults, Hispanics, Latinos, low-income, healthy aging, aging in place, independent living

## Abstract

**Background:**

Older Latino individuals are disproportionally affected by various chronic conditions including impairments in physical and cognitive functions, which are essential for healthy aging and independent living.

**Objective:**

This study aimed to evaluate the feasibility and acceptability of FITxOlder, a 12-week mind-body exercise program, in community-dwelling low-income, predominantly older Latino individuals, and assess its preliminary effects on health parameters relevant to healthy aging and independent living.

**Methods:**

This 12-week, single-arm, stage 1B feasibility study had a pre- and poststudy design. A total of 13 older adults (mean age 76.4, SD 7.9 years; 11/13, 85% Latino) of a congregate meal program in a senior center were enrolled. FITxOlder was a tailored Chinese mind-body exercise program using Five Animal Frolics led by a bilingual community health worker (CHW) participating twice a week at the senior center and facilitated by mobile health technology for practice at home, with incrementally increasing goals moving from once a week to at least 3 times a week. The feasibility and acceptability of the study were examined using both quantitative and qualitative data. Healthy aging–related outcomes (eg, physical and cognitive function) were assessed using paired 2-tailed *t* tests. Qualitative interview data were analyzed using thematic analysis.

**Results:**

The attendance rate for the 24 exercise sessions was high (22.7/24, 95%), ranging from 93% (1.8/2) to 97% (1.9/2) over the 12 weeks. Participants were compliant with the incremental weekly exercise goals, with 69.2% (9/13) and 75.0% (9/12) meeting the home and program goals in the last 4 weeks, respectively. Approximately 83% (10/12) to 92% (11/12) of the participants provided favorable feedback on survey questions regarding the study and program implementation, such as program content and support, delivery by the CHW, enjoyment and appeal of the Five Animal Frolics, study burden and incentives, and safety concerns. The qualitative interview data revealed that FITxOlder was well accepted; participants reported enjoyment and health benefits and the desire to continue to practice and share it with others. The 5-time sit-to-stand test (mean change at posttest assessment=−1.62; *P*<.001; Cohen *d*=0.97) and 12-Item Short Form Health Survey physical component scores (mean change at post intervention=5.71; *P*=.01; Cohen *d*=0.88) exhibited changes with large effect sizes from baseline to 12 weeks; the other parameters showed small or medium effect sizes.

**Conclusions:**

The research findings indicated that the CHW-led and mobile health–facilitated Chinese qigong exercise program is feasible and acceptable among low-income Latino older adults. The trending health benefits of the 12-week FITxOlder program suggest it is promising to promote physical activity engagement in underserved older populations to improve health outcomes for healthy aging and independent living. Future research with larger samples and longer interventions is warranted to assess the health benefits and suitability of FITxOlder.

## Introduction

### Background

Healthy aging is “the process of developing and maintaining the functional ability that enables well-being in older age” [1]. Being able to live independently is one of the key characteristics of healthy aging [2]. However, aging is accompanied by a variety of chronic conditions, and healthy aging is challenged by physical and cognitive function decline and, consequently, a lack of independence and compromised quality of life [3]. Approximately 75% of the diversity in the capacity and circumstances of healthy aging in older age results from the cumulative impact of advantages and disadvantages (eg, income and education) across people’s lives, and race and ethnicity are among the many contributing factors [1].

As the largest and most rapidly growing racial and ethnic minority in the United States [3,4], individuals aged ≥65 years of Latino or Hispanic origin have increasingly experienced health disparities in the aging process despite the noted Latino health paradox [5,6]. Older Latino individuals are more likely to have deteriorating physical and cognitive functions for independent living because of disabilities, poor mental health, and burdens of chronic disease from a lifetime exposure to poor conditions of daily life, poverty, discrimination, and substandard health care compared with their White counterparts [4,5,7,8]. This health disparity has contributed to longer durations of disabilities, a lower quality of life, and higher costs of health care in older Latino individuals while placing an increased burden on family members who provide their care during late life [4,5,7,8]. Despite urgent calls to develop culturally tailored and sustainable aging programs, research remains scarce on translating evidence-based practices to promote healthy aging in older Latino individuals who live in underserved communities [9-11].

As a distinctive form of complementary and alternative medicine [12], mind-body exercise refers to meditative movements that combine body movement, controlled breathing, and mental focus (or spirituality) to improve the attributes of physical fitness (strength, endurance, balance, and flexibility) and health-related outcomes [13,14]. Qigong, tai chi, and yoga are a few common forms of mind-body exercises that are rooted in Asian culture that have been accepted and practiced in Western countries in recent decades [15-17]. A well-established body of research using randomized controlled trials has demonstrated the clinically significant benefits of qigong, tai chi, and yoga on a variety of health outcomes, including but not limited to physical and cognitive function, quality of life, mental disorders, and chronic pain in diverse clinical and nonclinical populations in Western and non-Western countries [18-22]. Unlike traditional Western exercise, qigong exercise is characterized as low- to moderate-intensity, community-oriented with no or low cost, low-demand regarding space and equipment, and safe for all age groups and health conditions in Asian countries [22,23]. Recently, physicians in the United States and other Western countries have increased the use of the mind-body exercise for managing chronic health conditions and mental disorders following a holistic or integrative health framework. However, the uptake of mind-body exercises in Western countries is mostly limited to adults of younger age and higher socioeconomic status owing to limited availability and lack of awareness of health benefits compared with other forms of alternative health practices in the United States [16,17,24,25].

Although qigong and tai chi are often used interchangeably, qigong was generally used for the purpose of illness prevention and treatment by focusing on combining the physical and spiritual nature of the routines without the push for physical exhaustion, whereas tai chi had an origin in martial arts [26]. *Five Animal Frolics* or Five Animal Play (*Wu Qin Xi* in Chinese) is a traditional Chinese qigong that mimics the movements and spirit of a tiger, deer, bear, monkey, and crane to cultivate the energy emitted from the 5 animals and create harmony within all parts of the body and with the universe [26,27]. The playful and easy-to-learn movements of Five Animal Frolics can be practiced at different levels of physical exertion and range of movements to control the difficulty and stress loads of the exercise and, therefore, appeal to diverse groups, especially older adults [28].

Although evidence shows that qigong exercises can improve physical and cognitive function and quality of life and reduce fall risk, depression, and anxiety in older adults [21,22,29-32], methodological issues limit the scientific rigor and generalizability of the findings in older underserved racial and ethnic minority adults in the United States [22]. For example, most studies were conducted in China or with participants of higher socioeconomic status in Western countries. Another concern is the failure to address the spiritual, social, and cultural aspects of qigong exercise that can influence the uptake and maintenance of the mind-body exercises in older adults [28,33,34]. Although the concept of qi or energy is central in the teaching and practice of qigong, its meaning remains controversial among Western practitioners and researchers, and its acceptance has not been evaluated in studies conducted in Western populations [35]. This failure has contributed to the hesitation in recommending qigong and other mind-body exercises as part of holistic health care [36]. Finally, few studies have examined the barriers that limit the access to and availability and sustainability of mind-body exercise, such as transportation, family-friendliness, language, technology, and program cost in older underserved minority adults in the United States [33].

### Objectives

This paper reports the findings of a stage 1B feasibility pilot study [37] of a refined 12-week community-based and tailored Chinese qigong program using the Five Animal Frolics, “Function Improvement Exercises for Older Sedentary Community-Dwelling Latino Residents (FITxOlder),” to promote healthy aging in community-dwelling Latino older adults [38]. The primary purposes of the study were to (1) evaluate the feasibility of the intervention protocol in terms of program participation, fidelity of implementation, feasibility of the data collection protocol, progression of the stepped exercise program, and study-related adverse events and (2) examine the participants’ satisfaction with and feedback on the delivery of the 12-week program, cultural and age appropriateness of the intervention content and delivery, recruitment and retention practices, data collection protocol, and use of technologies for facilitating and supporting program delivery. The study also explored the changes in healthy aging–related outcomes in response to the exercise intervention.

## Methods

### Study Design and Sample

We conducted a single-arm stage 1B feasibility study with a pre- and posttest design to evaluate the feasibility and acceptability of FITxOlder, a 12-week healthy aging program, in older adults who were enrollees of a congregate meal program in a senior center in San Antonio, Texas. Most participants in the selected center were older Latino individuals. All the program participants were eligible to take part in the study if they were able to exercise in a standing position (with or without an assistive device), owned a cell phone, and agreed not to participate in other exercise programs. Those who planned to leave San Antonio during the 3-month study period were excluded. The senior center staff distributed the recruitment flyers to potential program participants. Those who were interested and met the study eligibility criteria attended an orientation session to learn the details of the study.

### Ethical Considerations

All participants completed a consent form to take part in the study. Recruitment information and consent forms were available in English and Spanish. The participants received up to US $90 (US $20 at baseline, US $30 at midtest assessment, and US $40 at posttest assessment) in grocery gift cards to compensate for their time for participation in data collection. The study protocol was approved by the University of Texas at San Antonio Institutional Review Board (protocol FY20-21-259).

### Intervention Program Description

FITxOlder was a community-based healthy aging program that was delivered by a trained community health worker (CHW) in a senior center with support and coordination from the senior center director. The FITxOlder intervention in this study was based on the protocol for a previous 3-arm feasibility study that explored the acceptance of qigong exercises and developed and piloted a healthy aging program using Five Animal Frolics among low-income Latino older adults. The original feasibility study included 2 phases: (1) planning and developing the intervention protocol based on input from the target population and (2) pilot-testing the feasibility and acceptability of the developed intervention protocol. The movement routines of Five Animal Frolics used in our study consisted of an opening routine, tiger routine 1 (raising tiger paws), tiger routine 2 (seizing the prey), deer routine 1 (colliding with the antlers), deer routine 2 (running like a deer), monkey routine 1 (lifting the monkey’s paws), monkey routine 2 (picking fruits), bear routine 1 (rotating the waist like a bear), bear routine 2 (swaying like a bear), crane routine 1 (stretching upward), crane routine 2 (flying like a crane), and closing routine. The routines are usually performed following a 13-minute narrated audio or video with background music embedded with animal sounds [27]. In the original pilot, one group learned the official version of Five Animal Frolics, the second group learned a modified version of Five Animal Frolics, and the third group received active control. The 4 components of the original FITxOlder were a CHW-led group session (didactic education, instruction based on the learning needs of older learners, and supervised practice), goal-based home practice (weekly exercise goals), a 3-stepped intervention with different instruction foci, and technology-facilitated support (tablet for showing the exercise video, SMS text message reminders, and follow-up phone calls), as described in Table 1. The weekly exercise goals increased from a minimum of 3 times a week to a minimum of 5 times a week by reaching an equivalent of ≥150 minutes of light to moderate physical activity (PA) per week. We also produced a 32-minute video (5-minute warm-up and replay of 13-minute movement routines of the official and modified Five Animal Frolics with a 1-minute break) to support and facilitate learning and practice at home. Unfortunately, the COVID-19 pandemic disrupted the implementation of the study protocol and prevented a full evaluation of the feasibility and acceptability of the pilot intervention. The rationale, development, and results of the original FITxOlder pilot program have been published elsewhere [38].

The protocol for the 12-week FITxOlder intervention in this study was refined using the findings from the original pilot intervention and poststudy participant focus group discussion to increase the program’s feasibility and acceptability. First, we aligned the conceptual framework of the intervention with the information-motivation-behavioral skills model that informed factors influencing the uptake and maintenance of a health practice (ie, participants’ learning and practice of Five Animal Frolics) [39]. FITxOlder provided the participants with knowledge of the background, healthy aging benefits, inner workings, and safety of qigong and Five Animal Frolics to increase their understanding and acceptance of the program. Motivation was facilitated by using a group format for social support, modeling by peers, and SMS text messaging and monitoring by a CHW and center staff. FITxOlder also focused on developing behavioral skills (goal setting and self-efficacy for practicing Five Animal Play) to engage the informed and motivated participants in the program and achieve the weekly exercise goals.

Second, the participants learned the modified Five Animal Frolics movements following the stepped instruction progression used in the original pilot (Table 1). In week 10, we introduced an advanced version of the modified Five Animal Frolics that increased the level of difficulty of the movements and physical exertion by increasing the range of movements and demand for balance (eg, on 1 foot). A community videographer made a new video of the modified Five Animal Frolics and a video of the advanced version of the routines for the study participants.

**Table 1:** Components of the FITxOlder program^a^.

Weeks	Intervention activities
1 to 4 (step 1)	Biweekly group sessions: attending two 60-minute group sessions led by a CHW^b^ each week Weekly home exercise goals: practice of Five Animal Frolics at least once at home following a video on a tablet Weekly program exercise goals: practice of Five Animal Frolics at least three times (twice at the center and at least once at home) Didactic education: introduction of Five Animal Frolics and qigong to the participants Instruction focus: teaching abdominal breathing; teaching choreography of the modified movement routines Participant support: weekly SMS text message reminder to perform the exercise at home and follow-up contact call by the CHW or senior center after missing a group session
5 to 8 (step 2)	Biweekly group sessions: attending two 60-minute group sessions led by a CHW each week Weekly home exercise goals: practice of Five Animal Frolics at least twice at home following a video on a tablet Weekly program exercise goals: practice of Five Animal Frolics at least four times (twice at the center and at least twice at home) Didactic education: visualizing the animals while performing the routine, mimicking the animals’ movements and spirituality, and discussing the correspondence between the movement routines and the health-related elements Instruction focus: teaching blending movements and breathing Participant support: weekly SMS text message reminder to perform the exercise at home and follow-up contact call by the CHW or senior center after missing a group session
9 to 12 (step 3)	Biweekly group sessions: attending two 60-minute group sessions led by a CHW each week Weekly home exercise goals: practice of Five Animal Frolics at least three times at home following a video on a tablet Weekly program exercise goals: practice of Five Animal Frolics at least five times (twice at the center and at least three times at home) Didactic education: teaching the consciousness of the present moment and energy, discussing the connections between the movements and the animals’ spirituality, and stressing the importance of combining the movements and breathing Instruction focus: teaching the blending of movements, breathing, and the animals’ spirit into “one” and teaching the advanced version of Five Animal Frolics Participant support: weekly SMS text message reminder to perform the exercise at home and follow-up contact call by the CHW or senior center after missing a group session

^a^The intervention protocol developed for the original pilot study.

^b^CHW: community health worker.

Third, in response to the feedback regarding the participants’ interest in learning the background of qigong, we decided to introduce basic information on qigong and Five Animal Frolics: the meaning of qi, history of Five Animal Frolics, symbolism (spiritual or cultural meanings) of the 5 animals, health-related elements associated with movement routines, and consciousness of the present moment. Exposing the participants to the basic background of qigong also allowed us to explore the appropriateness and acceptance of the cultural and spiritual aspects as part of qigong or Asian mind-body teaching.

Finally, we refined the group sessions to offer a supportive learning environment to promote engagement and a sense of mastery in the study participants who were older adult learners following the principles of geragogy [40-42]. The instruction strategies used by the CHW included (1) teaching the movement routine with a whole-part-whole learning model; (2) offering opportunities for active hands-on learning with repeated exposure to the content; (3) developing rapport with the participants, communicating with respect, and recognizing individual differences; (4) offering the background and reasons for learning a task at the early stage of learning; (5) promoting and supporting self-redirected and self-paced learning with respect to the participants’ life experience; and (6) providing regular feedback and focusing on small progress with positive reinforcement and support. The 60-minute session was divided into check-in and support, warm-up, supervised practice, instruction, and administrative time that allowed adequate time for participant engagement and support, as well as a minimum of 30 minutes dedicated to PA (Multimedia Appendix 1).

### Study Measures and Data Collection Procedures

There were 3 types of measures, which offered data to evaluate the feasibility and acceptability of the study, intervention protocols, and changes in healthy aging–related outcomes in response to the exercise intervention. Table 2 describes the measures and criteria used to evaluate the study feasibility. In addition, the CHW completed a group exercise session evaluation form to document reasons for missing the session, check the completion of planned class activities and participation, and report problems with lesson delivery at the end of each group session.

**Table 2:** Measures of feasibility.

Measure	Measurement procedure	Success criteria
Participant retention: completion rate of midtest and posttest assessment	Percentage of participants who completed the midtest and posttest assessment	≥80%
Participation: days attending the biweekly group sessions	Percentage of participants who attended the biweekly group sessions at the senior center (2 days per week) based on the attendance records kept by the CHW^a^	≥70%
Participation: days of practicing Five Animal Frolics using the 32-minute video at home following the weekly home exercise goals	Percentage of participants who achieved the weekly home exercise goals (≥1 day per week in weeks 1-4, ≥2 days per week in weeks 5-8, and ≥3 days per week in weeks 9-12) based on the reports from the weekly exercise logs kept by the participants	≥50%
Participation: total number of days of practicing the Five Animal Frolics exercise at the center and at home following the weekly program exercise goals	Percentage of participants who achieved the weekly exercise goals (≥3 days per week in weeks 1-4, ≥4 days per week in weeks 5-8, and ≥5 days per week in weeks 9-12) based on the attendance records and the weekly exercise logs	≥50%
Feasibility of the data collection protocol	Percentage of participants who completed all aging-related outcome measures at the baseline, 6-week, and 12-week assessments	80%
Study-related adverse events	Number of unanticipated adverse events that were related to the study in weeks 1 to 12 based on reports from the CHW and participants	None

^a^CHW: community health worker.

After completing the exercise in weeks 1 to 12, participants completed the Exercise-Induced Feeling Inventory to report feelings regarding revitalization, tranquility, positive engagement, and physical exhaustion as reactivity to the exercise on a 5-point scale from 0 to 4, where 0 stood for “do not feel at all” and 4 stood for “feel very strongly.” To document whether the meditative state of mind-body connection improved over time, participants completed a modified version of the Meditative Movement Inventory (MMI) [43] to report their perceived state in 2 dimensions (breath focus and meditative connection) on a 5-point scale (5 for *all the time*, 4 for *very frequently*, 3 for *occasionally rarely*, 2 for *very rarely*, and 1 for *never*) at weeks 2, 5, 7, 9, and 12. Higher scores indicated a higher level of meditative state. Finally, we created an observation form to evaluate the level of proficiency of the participants performing each routine of Five Animal Frolics in 3 dimensions (smooth and fluid movement, consistency of movement, and accurate imitation of movement) on a 5-point scale where 1 stood for *not at all*, 2 stood for *poor*, 3 stood for *fair*, 4 stood for *good*, and 5 stood for *excellent*. The evaluation was conducted by 2 observers monitoring 1 participant per routine who was selected randomly. The observations were repeated during the first and second rounds of the Five Animal Frolics exercise in 1 group session in weeks 5, 7, 9, and 12. The average of the scores from the 2 rounds was used. Higher scores indicated a higher level of proficiency.

To assess the acceptability of the intervention, the participants completed a poststudy survey to evaluate their satisfaction with the program content and delivery and provide feedback on the study protocol related to study burdens and incentives and exercise safety. Facilitation using digital technologies (using videos for modeling, tablets for showing the videos, and SMS text messages) was also evaluated. We expected that at least two-thirds of the participants would have a favorable response to the questions in the evaluation survey. We also conducted a focus group discussion with 7 study participants led by an English-Spanish bilingual facilitator to gather feedback on the delivery and content of the FITxOlder program 3 weeks after the completion of the study. The 7 participants were selected conveniently as whoever was available on a selected date when most participants could attend. The focus group discussion was guided by a predesigned interview guide, including topics such as *what challenges did you encounter when you practiced the exercise?* Probing questions were asked wherever appropriate. The discussion lasted approximately 60 minutes. The session was audio recorded and transcribed for analysis.

As part of the feasibility study, we collected data to explore the changes in healthy aging–related physical, cognitive, physiological, psychosocial, and behavioral outcomes in response to the exercise intervention (Table 3) at midtest assessment in week 6 and posttest assessment in week 13. The participants received written instructions 3 days in advance to prepare for the measurements. Trained bilingual research assistants and research faculty investigators conducted the physical (40 minutes), cognitive (30 minutes), and psychosocial (30 minutes) measurement of the participants in one 2-hour session at the senior center during the morning hours. All psychosocial measures were available in English and Spanish. At the end of the measurement session, the participants received a gift card for taking part in the data collection. The participants also received a report of their weight, blood pressure, and physical function tests over the 3 measurement time points at the end of the study.

**Table 3:** Description of outcome measures.

Outcome (healthy aging–related outcomes) and measure	Measurement procedure
Anthropometric measures and blood pressure	Height (cm) and weight (kg) were measured 2 times without shoes. The average was used. Systolic and diastolic blood pressure were measured 2 times after a 5-minute rest. The average was used.
Physical function	Physical function was assessed with a battery of physical function tests consisting of the 5XSTS^a^, 50-foot FWT^b^, hand grip test, 6MWT^c^, and FLRT^d^ [44].
Cognitive function	The Clock Drawing Test is a measure of visual-spatial abilities and cognitive function and has been used as a screen for cognitive impairment and dementia [45]. Participants are asked to draw the face of a clock with the hands set to “ten minutes past 11 o’clock.” Higher scores indicate high cognitive function. Performance on the Trail Making Test Part A and B is linked to visual search speed, speed of cognitive processing, mental flexibility, and executive functioning [46]. Participants are asked to draw a line to connect circles numbered 1 to 25 in ascending order in Part A and to connect the circles in an ascending order while alternating between numbers and letters (ie, 1-A-2-B-3-C) in Part B as quickly as possible. Longer time (seconds) to complete the task indicates lower level of cognitive function.
Psychosocial responses	Participants completed the SF-12^e^ [47] to generate a physical component score and mental component score of quality of life. Participants completed the BPI^f^ to report the level of perceived pain in 2 domains: pain severity (4 items) and pain interference with life (7 items) [48]. The participants completed the Sleep Disturbance Short Form 8a (8 items) [49] to report the quality of their sleep.
Behavioral response	Participants completed the basic ADL^g^ subscale (5 items) and intermediate ADL subscale (4 items) of the Functional Status Questionnaire [50]. The participants wore a GENEActiv triaxial accelerometer (Activinsights) on the nondominant wrist for 7 days. Time (hours per day) spent in sleep, sedentary activities, and light physical activity was estimated using the R package GGIR (version 1.10-7; R Foundation for Statistical Computing) [51] in the R environment (version 3.6.1).
Biomarker responses (data not reported)	A saliva sample was collected after a 12-hour fast. The participants also provided samples of saliva collected at home using a saliva collection kit.

^a^5XSTS: 5-time sit-to-stand test.

^b^FWT: fast walk test.

^c^6MWT: 6-minute walk test.

^d^FLRT: forward lean reach test.

^e^SF-12: 12-Item Short Form Health Survey.

^f^BPI: Brief Pain Inventory.

^g^ADL: activities of daily living.

### Data Analyses

The characteristics of the study participants were analyzed using descriptive analyses and are presented as means and SDs for continuous variables and percentages for categorical variables. We adopted descriptive statistics to assess the measures of feasibility and acceptability. Participants’ responses in the focus group discussion were processed using qualitative content analysis [52] to identify themes related to experiences of participating in the FITxOlder program. We also conducted a paired *t* test (2-tailed) to assess the responsiveness (ie, changes) in the healthy aging–related outcome variables from baseline to midtest and posttest assessments. The Cohen effect size coefficient (Cohen *d*) was used to evaluate the changes in each outcome (Cohen *d*=0.2 for small effect sizes, Cohen *d*=0.5 for medium effect sizes, and Cohen *d*=0.8 for large effect sizes) [53]. All analyses were conducted using R software (R Foundation for Statistical Computing) and SAS (version 9.4; SAS Institute).

The focus group discussion data were analyzed by researchers with extensive experience in qualitative analysis using a thematic analysis strategy. The principal investigator checked and verified the codes, and the study team discussed to categorize them into themes.

## Results

### Overview

A total of 13 participants (mean age 76.4, SD 7.9 years) aged between 62 and 86 years took part in the study (Table 4). The participants were primarily women (12/13, 92%), Latino (11/13, 85%), and English speakers (11/13, 85%). Almost all participants had a high school education or lower (12/13, 92%). All participants (13/13, 100%) met the low-income criteria for eligibility to the state and federal commodity supplemental programs. A total of 8% (1/13) of the participants used an assistance device for balance support, and another participant (1/13, 8%) received a diagnosis of Parkinson disease at the end of the study.

**Table 4:** Study participant characteristics (N=13).

Characteristic		Values
Age (years), mean (SD)		76.4 (7.9)
**Sex, n (%)**		
	Female	12 (92)
	Male	1 (8)
**Race and ethnicity, n (%)**		
	White	1 (8)
	Latino	11 (85)
	Black	1 (8)
**Preferred language, n (%)**		
	English	11 (85)
	Spanish	2 (15)
**Marital status, n (%)**		
	Married	3 (23)
	Not married	10 (77)
**Living arrangement, n (%)**		
	Lived with someone	3 (23)
	Lived alone	10 (77)
**Education, n (%)**		
	Completed elementary school	1 (8)
	Completed middle school	1 (8)
	Attended high school	3 (23)
	Completed high school or GED^a^	7 (54)
	Attended college or university	1 (8)
Income—qualified for the Commodity Supplemental Food Program because of low income, n (%)		13 (100)

^a^GED: General Educational Development.

### Feasibility Results

Retention of the participants was very high at the midtest and posttest assessments (Table 5). The completion rate of the data collection protocol for all the healthy aging–related outcomes was 100% (13/13) at baseline and >90% (12/13, 92%) at the midtest and posttest assessments. The attendance to the 24 biweekly group sessions remained high (22.7/24, 94%), ranging from 93% (1.8/2) to 97% (1.9/2) of sessions over the three 4-week periods. Participants were compliant with the incremental weekly home and program exercise goals, with 69.2% (9/13) and 75.0% (9/12) meeting the home and program goals in the last 4 weeks of the program, respectively. The fidelity of the lesson plan implementation was high, with 92% (33/36) of the sessions delivered as planned. The reasons for not completing the lesson plans were interruption because of technical problems with the television monitor and data collection activities. The level of feasibility of the assessed measures met the criteria of feasibility for the pilot study. There was no report of adverse events related to participation in the study. In all, 8% (1/13) of the participants withdrew from the study after the midtest assessment because of an unrelated, pre-existing health condition.

**Table 5:** Results of study feasibility (N=13).

Measurement procedure and time point		Value, n (%)
**Participants who took part in the assessments**		
	Midtest assessment	13 (100)
	Posttest assessment	12 (92)
**Participants who completed all aging-related outcome measures**		
	Baseline	13 (100)
	Midtest assessment	12 (92)
	Posttest assessment	11 (92)^a^
**Participants who attended the biweekly group sessions at the senior center**		
	Weeks 1-4	1.8 (93)
	Weeks 5-8	1.8 (93)
	Weeks 9-12	1.9 (97)
	Weeks 1-12	22.7 (94)
**Participants who achieved the weekly home practice goals (≥1 day per week in weeks 1-4, ≥2 days per week in weeks 5-8, and ≥3 days per week in weeks 9-12)**		
	Weeks 1-4	9 (75)^a^
	Weeks 5-8	7 (58)^a^
	Weeks 9-12	9 (69)
**Participants who achieved the weekly program exercise goals (center plus home: ≥3 days per week in weeks 1-4, ≥4 days per week in weeks 5-8, and ≥5 days per week in weeks 9-12)**		
	Weeks 1-4	10 (83)^a^
	Weeks 5-8	8 (67)^a^
	Weeks 9-12	9 (75)^a^
**Lessons in which the lesson activities were delivered as planned**		
	Weeks 1-4	7 (88)^b^
	Weeks 5-8	7 (88)^b^
	Weeks 9-12	7 (88)^b^
	Weeks 1-12	33 (92)^c^

^a^N=12.

^b^N=8.

^c^N=36.

**Figure 1:**
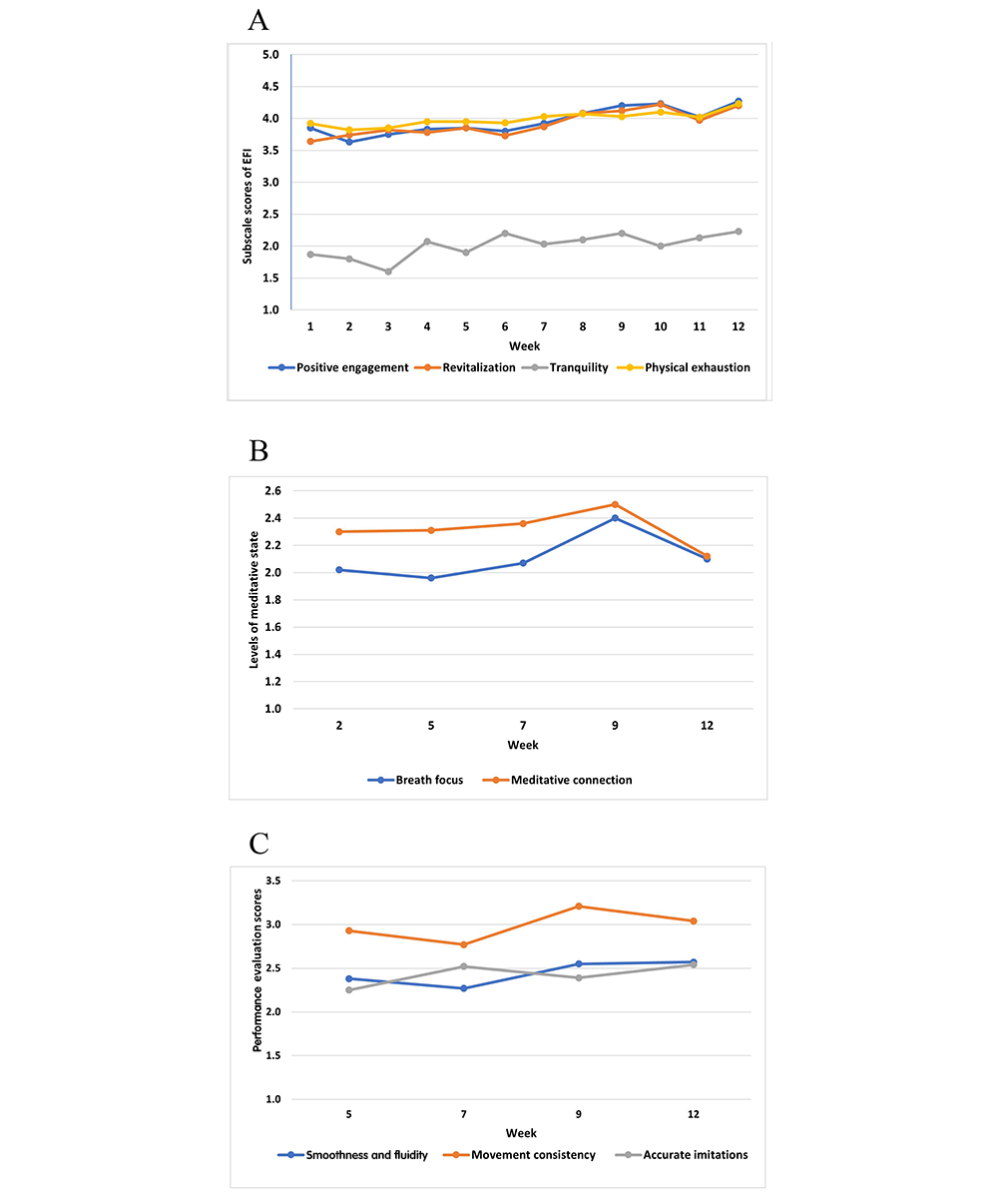
(A) Subscale scores (1-5) of the Exercise-Induced Feeling Inventory (EFI): positive engagement, revitalization, tranquility, and physical exhaustion from week 1 to week 12. (B) Levels of meditative state (1-5) over 5 time points for breath focus and meditative connection. (C) Five Animal Play performance evaluation scores (1-5) over 4 time points for smooth and fluid movement, movement consistency, and accurate imitations.

Figure 1A shows the modestly increasing trends in tranquility, revitalization, positive engagement, and physical exhaustion at the end of the exercise portion of each session over the 12-week period. The level of participants’ meditative state (ie, breath focus and meditative connections) measured by MMI increased gradually from week 2 to week 9 followed by a decrease in week 12 for both breath focus and meditative connection (Figure 1B). Figure 1C displays the modest upward trends of smooth and fluid movement, movement consistency, and accurate imitations of the Five Animal Frolics routines based on observations of performance by the participants in weeks 5, 7, 9, and 12.

### Acceptability Results

The level of satisfaction with the FITxOlder program was high, with a majority of participants providing favorable feedback on survey questions addressing the area of program content and support, delivery by CHW, enjoyment and appeal of Five Animal Frolics, opinions on incorporating the background information of qigong and Five Animal Frolics, study burden and incentives, and safety concerns, with a few exceptions (Multimedia Appendix 2). An overwhelming majority indicated high levels of acceptance of program content (10/11, 91%) and study incentives (11/11, 100%) and enjoyment of Five Animal Frolics (9/10, 90%-10/11, 91%) while expressing little concern for related safety (0%) and learning the cultural background of qigong and Five Animal Frolics (1/11, 9%). The participants were also very positive about the use of videos, tablets, and SMS text messages for facilitating program delivery and support. The participants also identified areas that could be improved (eg, the delivery of instruction and content by the CHW and long data collection sessions).

Identified themes and examples of corresponding quotes from the participants’ responses in the focus group discussion are presented in Multimedia Appendix 3. The most frequently mentioned theme was the *benefits associated with the program* (18 references), and the least frequently mentioned theme was *participation challenges due to family events and doctor’s appointments* (3 references). Overall, the FITxOlder program was well received and appreciated. The participants reported health, opportunity for PA, and socialization as motives and time conflicts and family commitments as barriers to program participation. The health benefits associated with the FITxOlder program included improvements in physical and mental health, pain, and sleep quality. The participants also expressed challenges when practicing Five Animal Frolics because of physical limitations or the complexity of the movements and routines but were able to modify (ie, body gestures) to accommodate them. Participants reported experiencing the spirit of the animals and acceptance of being taught qigong information in the instruction. Participants reported a preference for a model proximal to their age (rather than the younger model) in the Five Animal Frolics video, confusion regarding following the frontal or rear view of the model, and concerns about the timing of the cues in the narration. Finally, the participants reported having shared the exercise in their social circles and expressed a strong desire to continue the exercise in the future.

### Changes in Healthy Aging–Related Outcomes

The responsiveness of the healthy aging–related physiological, cognitive, psychosocial, and behavioral outcomes of the FITxOlder intervention is displayed in Table 6. There were 65% (15/23) of outcome measures that had noticeable differences from baseline to posttest assessment, including (1) changes with small effect sizes in systolic blood pressure, 5-foot fast walk, lean forward reach test, 6-minute walk test, Clock Drawing Test scores, Trail Making Test Part A scores, sleep disturbance, pain severity score, 12-Item Short Form Health Survey (SF-12) mental component scores, sedentary time, and light PA; (2) changes with medium effect sizes in pain interference scores and sleep time; and (3) changes with large effect sizes in the 5-time sit-to-stand test (mean change at posttest assessment=−1.62; *P*<.001; Cohen *d*=0.97) and SF-12 physical component scores (mean change at posttest assessment=5.71; *P*=.01; Cohen *d*=0.88). The changes from baseline to midtest assessment were smaller, as expected, but showed a favorable trend in 43% (10/23) of the outcome measures.

**Table 6:** Health outcomes at baseline, 6 weeks, and 12 weeks.

Variables		Baseline, mean (SD)	Midtest assessment, mean (SD)	Posttest assessment, mean (SD)	Baseline to midtest assessment			Baseline to posttest assessment	
					Change	Cohen *d*	Change		Cohen *d*
**Anthropometric measures and blood pressure**									
	Mean SBP^a^ (mm Hg)	149.96 (20.80)	139.5 (10.73)	142.75 (15.27)	−10.46	0.62	−7.21		0.39
	Mean DBP^b^ (mm Hg)	75.27 (11.88)	73.23 (8.92)	76.83 (14.24)	−2.04	0.58	1.56		0.12
	BMI (kg/m^2^)	30.13 (3.32)	30.80 (3.68)	29.99 (4.35)	0.66^c^	0.19	−0.15		0.04
	Body fat percentage, %	41.22 (4.81)	41.57 (4.7)	41.46 (5.6)	0.35	0.07	0.24		0.03
**Physical function**									
	5XSTS^d^ (seconds)	12.68 (2.45)	11.93 (3.59)	10.74 (2.21)	−0.75	0.24	−1.62^c^		0.97
	Grip strength (dominant hand; kg)	19.10 (6.82)	18.45 (5.8)	19.61 (5.96)	−0.65	0.23	0.11		0.05
	FWT^e^ (seconds)	16.00 (5.03)	18.82 (9.82)	15.06 (4.73)	2.82	0.36	−0.94		0.20
	Reach (cm)	25.35 (6.55)	22.75 (9.24)	27.42 (4.57)	2.6	0.33	2.06		0.36
	6MWT^f^ (m)	383.28 (116.67)	392.56 (130.32)	412.02 (118.72)	9.28	0.08	28.73		0.24
**Cognitive function**									
	Clock Drawing Test score	7.46 (1.98)	8.62 (1.26)	8.08 (1.68)	1.15^g^	0.69	0.62		0.24
	TMT^h^ Part A (seconds)	73.14 (68.09)	62.89 (61.12)	58.23 (54.19)	−10.26	0.16	−14.91		0.24
	TMT Part B (seconds)	161.72 (107.66)	166.89 (161.33)	149.87 (166.44)	5.17	0.04	−11.84		0.09
**Psychosocial and behavioral measures**									
	Sleep disturbance score	23.38 (2.53)	23.23 (2.59)	22.67 (2.74)	−0.15	0.06	−0.72		0.27
	Pain severity score	2.83 (1.92)	2.52 (2.85)	2.35 (1.87)	−0.31	0.13	−0.47		0.25
	Pain interference score	2.89 (2.64)	2.23 (2.37)	1.47 (1.72)	−0.66	0.26	1.42		0.64
	Basic ADL^i^ score	10.69 (3.28)	11.31 (1.03)	10.67 (3.42)	0.62	0.25	0.03		0.01
	Intermediate ADL score	18.62 (5.42)	18.92 (3.97)	18.92 (3.70)	0.31	0.07	0.31		0.06
	FSQ^j^ total score	29.31 (8.02)	30.23 (4.21)	29.58 (6.4)	0.92	0.14	0.28		0.04
	SF-12-PCS^k^ score	39.15 (9)	42.78 (9.75)	46.43 (8.6)	3.64	0.39	5.71^g^		0.88
	SF-12-MCS^l^ score	53.80 (9.87)	53.87 (12.03)	50.57 (11.06)	0.07	0.01	−3.23		0.30
	Sleep (hours per day)	6.29 (1.36)	6.30 (1.49)	6.83 (2.49)	0	0	0.53		0.52
	Sedentary time (hours per day)	13.92 (2.28)	13.85 (2.23)	12.92 (3.35)	−0.07	0.03	−0.99		0.35
	LPA^m^ (hours per day)	2.78 (1.39)	2.89 (1.04)	3.26 (1.12)	0.1	0.1	0.47		0.37

^a^SBP: systolic blood pressure.

^b^DBP: diastolic blood pressure.

^c^*P*<.01.

^d^5XSTS: 5-time sit-to-stand test.

^e^FWT: 50-foot fast walk test.

^f^6MWT: 6-minute walk test.

^g^*P*=.01.

^h^TMT: Trail Making Test.

^i^ADL: activities of daily living.

^j^FSQ: Functional Status Questionnaire.

^k^SF-12-PCS: 12-Item Short Form Health Survey physical component score.

^l^SF-12-MCS: 12-Item Short Form Health Survey mental component score.

^m^LPA: light physical activity.

## Discussion

### Principal Findings

The results of the study support the feasibility and acceptability of the refined FITxOlder, a community-based mind-body exercise program based on the Five Animal Frolics delivered by a CHW with technology-facilitated support and tailored for low-income community-dwelling Latino older adults. Strong feasibility was demonstrated by participant retention and program attendance; compliance with exercise goals was indicative of the increased appeal of complementary and alternative medicine and mind-body exercise [54] for healthy aging in older adults who are seeking a socially, culturally, and geragogically tailored program [33,55,56]. The trending health benefits in physical function, cognition, and psychosocial well-being as well as objective measures of PA and sleep are essential for healthy aging and independent living [2,3].

### Feasibility and Acceptability

The high level of engagement and retention in this study can be partially attributed to the engagement of older adults in developing the PA program [57]. FITxOlder was based on lessons learned from a previous pilot study that used an iterative process to gather input from a work group of Latino older adults in developing and tailoring the delivery of the FITxOlder program as well as the Five Animal Frolics routines [38]. For example, participants were appreciative of the group format that offered opportunities for socialization and peer support in FITxOlder [58,59] and the program delivery by a bilingual CHW who provided linguistically and socially sensitive support to engage the participants [60]. The findings also reflect older adults’ interest in improving quality of life and independent living beyond health indexes [61]. In addition, FITxOlder was designed as a safe and low to moderate intensity mind-body exercise, which was favored by older adults with considerable health conditions that hindered them from engaging in intensive exercise programs [62]. However, feedback from the participants revealed needs for improvement in the quality and design of the exercise video and instruction delivery. Finally, the use of the exercise video modeled by the CHW and of SMS text message reminders facilitated the participation in home practice and the achievement of weekly exercise goals. The overall adherence to the prescribed exercise frequency in the study was similar to the adherence rate in published exercise studies on older adults [63].

The upward trend in tranquility, revitalization, and positive engagement scores of the Exercise-Induced Feeling Inventory subscales reflected positive psychological and emotional reactions in the participants that have been associated with increased PA level as well as exercise adherence in previously published studies on adults [64,65] and older adults [66,67]. Participants in a 10-week yoga intervention group also demonstrated increased tranquility, revitalization, and positive engagement [68]. Furthermore, it is worth pointing out that the scores on the physical exhaustion subscale at each group session consistently exceeded the level reported by older adults who completed a 30-minute moderate-intensity aerobic exercise routine (≥64% of maximum heart rate) [66].

The gradual increases in the scores of breath focus and meditative connection on the MMI and in the observed movement smoothness and fluidity and accuracy of movement imitation were consistent with the progressive goals in the 3 steps of the instructions (ie, the participants’ meditative state and movement proficiency improved with in-class instruction and practice during the first 9 weeks [43]). The decline in the scores in week 12 was the result of introducing the advanced version of the Five Animal Frolics that started in week 10. Although it is normal to experience reduced mastery when a more challenging routine is introduced, the decline in the scores indicated that the participants were inadequately prepared to learn the advanced version, and thus their experience of mastery was adversely affected. However, the overall changes in scores suggest that the participants were responsive to the stepped instruction and support the validity of the MMI and performance observation.

The refined FITxOlder was highly acceptable to the study participants, as demonstrated by the high retention, high program attendance, and compliance with weekly exercise goals by FITxOlder participants. Our results were consistent with the findings of a 2021 systematic review that reported high attendance, with 73% to 95% participation in supervised group sessions and adherence to home self-practice (63%-80%) in women taking part in qigong interventions [69]. However, past healthy aging studies using traditional Western exercise or tai chi have reported low retention and low program participation because of dissatisfaction with the program delivery format or the program not meeting the participants’ needs (disease focus vs holistic health) [70,71]. For example, HAPPY, a community-based healthy aging program of biweekly group sessions delivered by a peer coach using traditional Western exercise, significantly improved physical and cognitive functions but only retained 66.6% of older adult participants at the 3-month follow-up [72]. Furthermore, results from our quantitative and qualitative analysis indicated that the Five Animal Frolics being easy to learn, suitable for home practice, and safe. The acceptance of using a tablet for FITxOlder practice suggests that Latino older adults can benefit from using digital technologies provided by the study for health promotion practices [73,74]. A previous study reported that older participants were able to practice qigong at home using a study-provided video [75]. FITxOlder participants also expressed the importance of SMS text message reminders for keeping them on track to meet weekly exercise goals. Of note, the percentage of participants who achieved the weekly home practice goals was not optimal and was lower than the rate of attendance to group sessions. This suggests that the at-home practice recommendation did help increase exercise engagement to some extent; it also suggests the importance of a supportive learning environment to promote exercise engagement. The completion rate (11/12, 91.7%-13/13, 100%) of data collection was higher than those reported in past qigong studies (67%) [69], and most participants (9/10, 90%) did not consider the data collection burdensome. Finally, this study offered important insight on the acceptance of introducing background information on qigong and Five Animal Frolics in a predominantly Latino sample. In the future, it is important to further explore the best approach for the integration of the multidimensions of qigong into the holistic health framework for effective dissemination to non-Asian population groups [76,77].

### Healthy Aging–Related Outcomes

Despite the light to moderate intensity of the Five Animal Frolics, the responsiveness in the healthy aging–related outcomes was promising and showed an effect size consistent with published randomized controlled trials in older adults with longer intervention durations using traditional Western exercises [78], tai chi [79,80], or Five Animal Frolics [81,82]. Of particular interest were the changes in the 5-time sit-to-stand and SF-12 physical component scores, which were reflective of improvements in physical functions for independent living among older adults [83,84]. To be noted, there was also an improvement in cognitive function, another key factor for healthy aging and independent living [85,86]. By contrast, the decline in SF-12 mental component scores from midtest to posttest assessments was counterintuitive to the reported socialization benefit from participating in the FITxOlder program. We speculate that worsening health conditions (ie, diagnosis of mental disorder and surgery) unrelated to the study and family hardships in some of the participants in the last 4 weeks of the intervention might have contributed to the decline in mental health. Finally, the slightly increased BMI from baseline to midtest assessment was unexpected. On the basis of the report of a review, overall studies directly examining the effects of mind-body exercise on body weight are limited [87]. A recent study randomized 543 participants into a tai chi group, a conventional exercise group, and a control group and reported reduced body weight and waist circumference in both intervention groups but not in the control group at weeks 12 and 38 [88]. The midtest assessment in our study at 6 weeks after the intervention may not have been long enough to show exercise effects. Particularly, our study did not consider other factors such as diet intake, which are also important for body weight and BMI.

There was indirect evidence that the FITxOlder program helped participants meet PA recommendations [89] as participants practiced ≥30 minutes per day of Five Animal Frolics on most days of the week during the last month of the intervention. The increase in PA was corroborated by objectively measured changes in time spent in light PA (+28.2 minutes per day) as well as sedentary time (−59.4 minutes per day). Of note, the increase in light PA is particularly important as Five Animal Frolics consists of slow and smooth movements that will be recorded as light rather than moderate PA by accelerometry. The increase in sleep duration (+38.3 minutes per day) was another benefit for maintaining physical and cognitive functioning in older adults [90].

### Limitations

There are several limitations to this study. The changes observed in the study should be interpreted with caution because of the short intervention duration and use of a single-arm design. Previous studies have reported significant improvements in the outcomes of older adults using interventions lasting ≥6 months [91]. The single-arm design also did not allow for the testing of a placebo effect associated with within-study socialization and attention received by the study staff [92]. The efficacy of qigong for healthy aging should be tested using a rigorous study design, a larger sample size, and long-term follow-up. In addition, the improvement in physical and cognitive function outcomes may be due to a learning effect caused by repeated testing. Not all the participants in this study were Latino. However, all participants (13/13, 100%) were older adults from the same low-income community, and most of them (11/13, 85%) were Latino. Therefore, our findings could still be applicable to our target population of low-income community-dwelling older Latino individuals. Finally, most of the study participants (12/13, 92%) were women. The generalizability of the findings to men is limited. Strategies for recruiting male participants to such studies need to be explored.

### Conclusions

Healthy aging research in underserved minority populations remains limited [93]. The FITxOlder program demonstrated high feasibility and acceptability for both in-person delivery and practice at home among low-income Latino older adults. The trending health benefits of this short-term intervention suggest that the FITxOlder program might be a promising approach to promote PA engagement in underserved older populations for various health improvements (eg, physical and cognitive function and psychosocial well-being), which are essential for the promotion of healthy aging and independent living. Future research with larger samples and longer interventions is warranted to assess the health benefits of the FITxOlder program and its suitability.

## Appendix

Multimedia Appendix 1 Biweekly group session format.

Multimedia Appendix 2 The 12-week older adult exercise study poststudy survey.

Multimedia Appendix 3 Themes from the focus group discussion.

## Abbreviations

CHW: community health worker
MMI: Meditative Movement Inventory
PA: physical activity
SF-12: 12-Item Short Form Health Survey
