# Supplementary material for: Examining the Delivery of a Tailored Chinese Mind-Body Exercise to Low-Income Community-Dwelling Older Latino Individuals for Healthy Aging: Feasibility and Acceptability Study
Source: JMIR Form Res. 2022 Sep 13;6(9):e40046. doi: 10.2196/40046 (PMC9516366; doi:10.2196/40046)
Supplement: Multimedia Appendix 1 [file formative_v6i9e40046_app1.docx]

Multimedia Appendix 1

Table S1. Bi-weekly Group Session Format.

| Class Activity | Time |
| --- | --- |
| Meet-and-greet (Repeated during all sessions)   - Taking attendance - Sharing of experience from previous session (Questions, and comments) - Checking for adverse event | 5-10 minutes |
| Warm-up (Repeated during all sessions)   - General light Warm Up including basic movements of Five Animal Frolics focusing on appropriate form, controlled movements, and abdominal breathing. | 5 minutes |
| Instructor-led practice of Five Animal Frolics following a video^a^ displayed on a large-screen TV monitor | 13 minutes |
| Instruction of Five Animal Frolics movement routines^b^   - Didactic education - Teaching and demonstrating a movement subroutine   - Explaining isolating movements (upper body, lower body, hand, arm, etcetera)   - Relating movements with animal spirit   - Posture and breathing - Active, hands-on learning | 10 minutes |
| Instructor-led practice of Five Animal Frolics following a video displayed on a large-screen TV monitor | 13 minutes |
| Closing remarks   - Completion of exercise feeling survey and exercise log - Reinforcement of home exercise goals - Problem-solving on technical issues related to Five Animal Frolics | 5-10 minutes |

^a^ A 13-minute video of the modified Five Animal Play was used in weeks 1-8; an advanced version of the modified movements was used in weeks 9-12.

^b^ CHW usually focused on one movement routine per session.
