# Supplementary material for: Examining the Delivery of a Tailored Chinese Mind-Body Exercise to Low-Income Community-Dwelling Older Latino Individuals for Healthy Aging: Feasibility and Acceptability Study
Source: JMIR Form Res. 2022 Sep 13;6(9):e40046. doi: 10.2196/40046 (PMC9516366; doi:10.2196/40046)
Supplement: Multimedia Appendix 2 [file formative_v6i9e40046_app2.docx]

Multimedia Appendix 2

Table S2. 12-week Senior Exercise Study Post Study Survey^a^

|  | Strongly agree  **n (%)** | Agree  **n (%)** | Disagree  **n (%)** | Strongly disagree  **n (%)** |
| --- | --- | --- | --- | --- |
| **Evaluation of program content and support** | | | | |
| The time of doing the Five Animal Frolics exercise in group sessions at the senior center were appropriate for me (n=10). | 5 (50%) | 4 (40%) | - | 1 (10%) |
| I wish we had more time to do the exercises during the group sessions at the senior center (n=9). | 1 (11%) | 5 (56%) | 2 (22%) | 1 (11%) |
| The narrated Five Animal Frolics videos helped me to learn the exercise movements (n=11). | 4 (36%) | 6 (55%) | - | 1 (9%) |
| The narrated Five Animal Frolics videos helped me to do the exercise at home (n=11). | 4 (36%) | 6 (55%) | - | 1 (9%) |
| The tablet from the study helped me to do the exercise at home (n=11). | 4 (36%) | 5 (45%) | - | 2 (18%) |
| A tablet with a larger screen would be more helpful to learn and practice the Five Animal Frolics (n=10). | - | 1 (10%) | 5 (50%) | 4 (40%) |
| The phone text messages encouraged me to meet the exercise goals (n=7). | 2 (29%) | 4 (57%) | - | 1 (14%) |
| **Content delivery by CHW** | | | | |
| The exercise instructor gave clear verbal instructions on how to do the movements of the five animals in the group setting in the senior center (n=11). | 5 (45%) | 3 (27%) | 1 (9.1%) | 2 (18%) |
| The exercise instructor gave clear demonstrations on how to do the movements of the five animals in the group setting in the senior center (n=10). | 5 (50%) | 3 (30%) | 1 (10%) | 1 (10%) |
| The exercise instructor clearly explained the health benefits of Chinese Health Qigong in the group setting in the senior center (n=11). | 3 (27%) | 6 (55%) | 1 (9%) | 1 (9%) |
| The exercise instructor clearly explained the cultural background of Chinese Health Qigong and the Five Animal Frolics in the group setting in the senior center (n=11). | 5 (45%) | 4 (36%) | 1 (9%) | 1 (9%) |
| **Enjoyment of Five Animal Frolics** | | | | |
| I enjoyed doing the Five Animal Frolics exercise (n=11). | 4 (36%) | 6 (55%) | - | 1 (9%) |
| I felt better after I started doing the Five Animal Frolics exercise (n=10). | 3 (30%) | 6 (60%) | - | 1 (10%) |
| I felt stronger after I started doing the Five Animal Frolics exercise (n=11). | 2 (18%) | 8 (73%) | - | 1 (9%) |
| Doing the Five Animal Frolics exercise has become an important part of my daily life (n=10). | 2 (20%) | 7 (70%) | - | 1 (10%) |
| **Evaluation of the appeal of Five Animal Frolics** | | | | |
| I would recommend Five Animal Frolics exercises to other folks like me (n=11). | 5 (45%) | 5 (45%) | - | 1 (9%) |
| I asked others to do the Five Animal Frolics with me (n=11). | 2 (18%) | 2 (18%) | 5 (45%) | 2 (18%) |
| I showed others how I did my Five Animal Frolics (n=10). | 4 (40%) | 3 (30%) | 2 (20%) | 1 (10%) |
| I will continue doing the Five Animal Frolics exercise after the study ends (n=10). | 3 (30%) | 6 (60%) | - | 1 (10%) |
| I will do the Five Animal Frolics exercise if the group sessions continue in the senior center (n=10). | 3 (30%) | 6 (60%) | - | 1 (10%) |
| **Feedback on introduction of background information of Qigong and Five Animal Frolics** | | | | |
| Learning the health benefits of Chinese Health Qigong motivated me to do the Five Animal Frolics exercise (n=10). | 3 (30%) | 6 (60%) | - | 1 (10%) |
| Learning the cultural background of Chinese Health Qigong helped me to learn the exercise movements of the Five Animal Frolics (n=10). | 2 (20%) | 7 (70%) | - | 1 (10%) |
| I did not like the discussion of the cultural background of Chinese Health Qigong and the Five Animal Frolics (n=11). | - | 1 (9%) | 4 (36%) | 6 (55%) |
| **Evaluation of study burdens and incentives** | | | | |
| We spent too much time in data collection throughout the study (n=10). | - | 1 (10%) | 7 (70%) | 2 (20%) |
| I did not mind participating in the tests and surveys in the beginning, middle and end of the study (n=11). | 4 (36%) | 4 (36%) | 1 (9%) | 2 (18%) |
| The biggest reason I participated in the data collection was because of the HEB gift cards (n=11). | 1 (9%) | - | 5 (45%) | 5 (45%) |
| I think the gift card amount for data collection was not enough (n=11). | - | - | 6 (55%) | 5 (45%) |
| **Assessment of safety concerns** | | | | |
| I was worried about getting injured when I did the Five Animal Frolics exercise in the group sessions in the center (n=10). | - | - | 5 (50%) | 5 (50%) |
| I was worried about getting injured when I did the Five Animal Frolics exercise at my home (n=10). | - | - | 5 (50%) | 5 (50%) |
| ^a^ Some participants did not complete all of the questions. | | | | |
