# Supplementary material for: Examining the Delivery of a Tailored Chinese Mind-Body Exercise to Low-Income Community-Dwelling Older Latino Individuals for Healthy Aging: Feasibility and Acceptability Study
Source: JMIR Form Res. 2022 Sep 13;6(9):e40046. doi: 10.2196/40046 (PMC9516366; doi:10.2196/40046)
Supplement: Multimedia Appendix 3 [file formative_v6i9e40046_app3.docx]

Multimedia Appendix 3

Table S3. Themes from the focus group discussion.

| Theme | Number of references | Quotes (examples) |
| --- | --- | --- |
| Motives for participation | 8 | “…friendship too that we you know like I said I just retired I just didn't want to be at home you know feeling depressed…”  “I was told by my physicians that I needed to get out and be more active and socialize more”  “I was gaining weight and the more I weigh the more pain I feel so I wanted to do something, and I did I don't have anything against gyms and all those Planet Fitness stuff it's it's just that it every time I would go there I felt so awkward I didn't feel like I belong there. now here it's like hey were all were all great here we’re all here you know.” |
| Participation challenges due to family events and doctor appointment | 3 | “my challenge was that the first day I started I got a call that one of my nephews had passed away and then it seemed like every time something was happening with you it would seem like I was bomb barded with my niece calling me and telling me that the doctors told her we can’t do anything for you anymore and you know stuff like that…” |
| Benefits associated with program | 18 | “I don't sleep at nights well now I'm sleeping much much better. So this experience to me it's helped me out not only mentally, physically, you know it's helped me so much in the sense, that I lost, in the 12 weeks, I lost about four pounds.”  “I'm always in pain so I rather be here doing this, and I do them at home...”  “…so, the Qigong helped with our physical and our mental status but it's also more the fellowship...”  “my husband won’t do the exercises with me…but he liked what it did to me not only physically but he said…you’re smiling more…” |
| Difficulty in performing and mastering the routines due to physical challenges and movement/routine complexity | 8 | “my hands lock up…so I wasn’t always able to as we were instructed to or as we saw in the screen”  “Even if we can’t do it right or we’re slow or fast, we still do the best we can, and it helps a lot”  “I get disgusted when I can’t do a step…it doesn’t matter but you still feel like guilt”.  “And my, for bird, because of my arm I couldn't you know … I couldn't even pick up my arm and now I’m doing good” |
| Modification of the exercise routines to accommodate health conditions and safety concerns | 6 | “I kept doing mine with holding on to the chair. either with one or both hands and that is that helped me a lot 'cause I was afraid to fall down even now I still hold on to the chair.”  “…because people our age, you know, our joints hurt, we’re afraid of falling down…” |
| Feedback on the production of the exercise videos | 8 | “…to see somebody who's older not a young person that's going with somebody that's older you can see that how they do it and you can feel like well OK well maybe that person can do it you know maybe it's OK and I can do it too.”  “…it was one side one side was not in sync with the other and then you know so and then the voice also it was already well put your hands up and on the tv…” “…It’s still not in sync…” |
| Understanding of Qigong and Five Animal Frolics  Accepting Five Animal Frolics as a form of exercise without regard to its origin | 10 | “Exercise is an exercise, …hey, it felt good”  “…center of uh, center of something I don't know what I know it was center of something…” |
| Experiencing the spirit imbedded in the animals | 8 | “…when I would do the bird, it would transform me. I felt like I was flying…just gliding down, that’s what I love”  “(the monkey). It was an easier movement for my body I could do that one with ease I didn't have to make an effort to do it” |
| Sharing Five Animal Frolics with others | 5 | “I'm also doing the Qigong with the ladies at my church. I'm they they said they saw a difference in me right away they said they saw glow in me, they said ‘you look fit’”.  “I told them stuff that I do and and so they told me what demonstrate so I started them and stuff so and stuff so so now we don't we do it once a month…” |
| Desire to continue practice of Five Animal Frolics | 9 | OK once we’re done doing it here…I'm doing it till I die and hopefully it won't be soon, hopefully, but we'll see. |
